# Hydropower generation by transpiration from microporous alumina

**DOI:** 10.1038/s41598-021-90374-5

**Published:** 2021-05-26

**Authors:** Manpreet Kaur, Satoshi Ishii, Ryusuke Nozaki, Tadaaki Nagao

**Affiliations:** 1 International Center for Materials Nanoarchitectonics (WPI-MANA), National Institute for Materials Science (NIMS), Tsukuba, Ibaraki 305-0044 Japan; 2 Department of Condensed Matter Physics, Hokkaido University, Sapporo, Hokkaido, 060-0810 Japan

**Keywords:** Materials for devices, Energy science and technology, Energy harvesting, Devices for energy harvesting

## Abstract

Traditional hydropower generation is one of the most sustainable energy sources; however, the local environmental impact of hydroelectric dams and reservoirs is serious, and hydroelectric power requires high-cost turbines and generators. Because these installations utilize gravitational potential energy of massive volumes of falling water, this sort of hydropower generation is unsuitable for ubiquitous, small-scale energy production. Here, we report that wetting and evaporation of pure water from a tiny block of porous alumina generates electrical current in the direction of water transpiration. The current induced in microporous alumina is associated with mass transport of water accompanying ions that accumulate near the negatively charged surface of alumina pores. Without any pre-treatment or additives, once evaporation commences, a 3 × 3 cm^2^ piece of alumina can generate an open-circuit voltage as large as 0.27 V. The power generation scheme we propose here is simple, clean, and versatile, and it can be employed anywhere, as it utilizes only spontaneous capillary action of water and Coulombic interaction at the alumina-water interface, without requiring any input of heat or light.

## Introduction

Development of renewable energy sources with practically zero carbon emission has become increasingly important^1–3^. Various efforts have attempted to convert ubiquitous natural energy to electricity, using solar cells^4, 5^, thermoelectric^6, 7^ and piezoelectric/triboelectric generators^8^, as well as nano electric generators^8, 9^. As seen from these examples, generating sustainable and stable electricity from natural sources usually requires sophisticated hetero-structured materials and complex device configurations^10–13^. Hydropower generation produces about one-sixth of global electricity without the cost of carbon emission, but it requires large-scale construction with heavy impact on natural environments and it requires sophisticated machinery^14^. Recently, evaporation-induced power generation has attracted great interest as a miniaturized hydropower source that mimics transpiration of water in plants^15–18^. The electricity generated originates from the streaming current of electrolytes, where movements of ions near the surface of a conducting substrate induce a voltage drop associated with movement of water molecules. The presence of an electrical double layer (EDL) at the interface between the liquid and porous wall forms the basis of charge movement during water evaporation^19–21^.

In early demonstrations, small electrical signals were detected when CNTs were immersed in flowing water or polar liquids^22–26^. However, the observed voltage was only on the order of microvolts/cm, and fabrication and handling of CNTs are incompatible with large-scale applications. Similarly, a few millivolts can be generated using carbon black^20^, graphene oxide^27, 28^, monolayer graphene^29, 30^, as well as carbon-based hybrid systems grown on semiconductor nanowire networks^31^ or cellulose-based filter paper printed with multi-walled CNTs^32, 33^. However, most of these carbon-based materials adhere only weakly to substrates, making devices fragile and hard to scale up, and the output voltage is usually below 100 mV/cm^2^^22, 27, 29, 30, 34, 35^. Recently, several dielectric samples were explored in order to harvest electricity from water evaporation, based on its streaming potential. These included natural wood^36^ and oxide nanoparticle-based flexible hydroelectric film^37, 38^, increasing the variety of possible device structures and the feasibility of this approach for various applications.

In this study, we discovered that substantial voltage generation is possible across an alumina “*insulator*” block soaked in deionized water. Here we propose that compared to carbon-based devices, microporous alumina driven by water transpiration is an efficient, prototypical, power-generating substrate to achieve high voltage efficiency. Micro/nano-porous alumina has a long history of applications in optical, chemical, and biological sciences and relevant engineering fields^39–42^. The surface chemistry of alumina is essential to its performance as a building block for biosensing^43^, water desalination^44^, and nanoelectronic devices^45^, as it is chemically stable in aqueous environments and at high temperatures^46, 47^. In this work, we report the use of robust porous alumina without any pre-treatment or coating for electrical generation driven by water evapotranspiration. Its porosity drives the capillary force and transpiration through the block, resulting in power generation. A 3.0 × 3.0 cm^2^ block shows the capacity to generate an open circuit potential as high as ∼0.27 V with a stable power-generating performance exceeding a year under ambient conditions. Among different conditions investigated in our work, the maximum streaming voltage was obtained when one-half of the porous alumina sample was partly soaked in water at an appropriate placement angle. Output power can be flexibly tuned by changing the wetting conditions of the porous medium, water temperature, and salt concentration, as well as by simply altering connections of the modules in series/parallel. We believe that abundant interconnected pores provide a large dielectric (polarized) alumina surface that produces sufficient charge to yield effective carrier diffusion at the water-alumina interface. This phenomenon is based on evapotranspiration of water and does not require any supply of light or heat. This indicates that the proposed energy harvesting method can work anywhere on earth, any time of day, making it a true energy-harvesting device. Such electricity generators inaugurate a robust and facile energy-harvesting method, applicable to small-scale power generators for self-powered sensor networks, as well as electric generators that can operate on cloudy days and at night.

## Experimental

**Figure 1:**
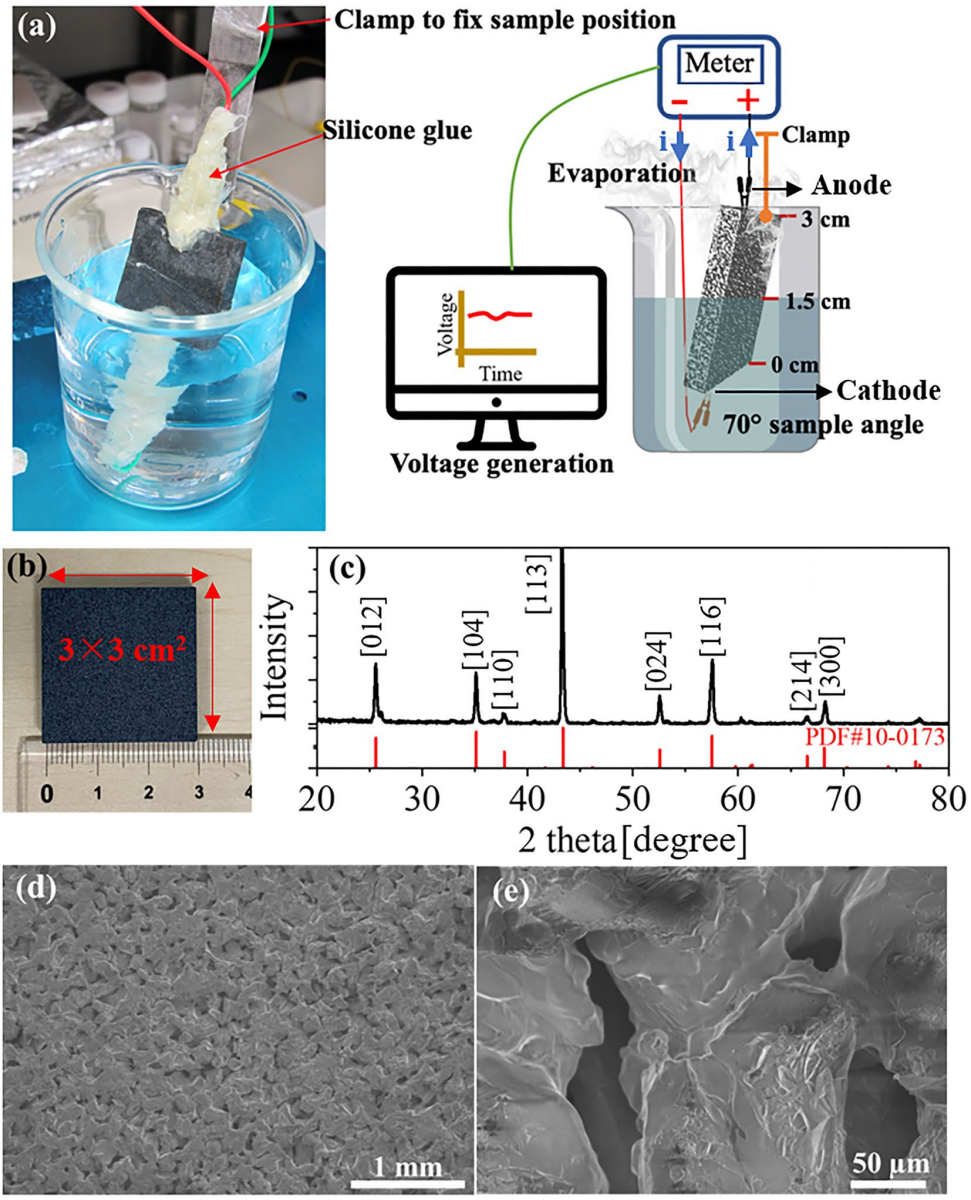
(**a**) Schematic diagram of the experimental setup and a photograph of the device. (**b**) Photograph of the porous alumina block. (**c**) XRD pattern (black curve) of the alumina and a pattern from the PDF card (10-0173) (red). (**d**) SEM image of alumina. (**e**) Magnified view of (**d**).

Alumina with ~ 35% porosity was purchased from ASUZAC Fine Ceramics. Its chemical composition was analysed by energy-dispersive X-ray spectroscopy (EDX). Sample morphology was examined by SEM (Hitachi FE-SEM SU8230). X-ray diffractions were taken using a Rigaku Ultima III, Rint 2000. Electrical contact with the alumina was achieved using conductive clamps. Silicone glue was used to prevent a possible electrical short circuit and a clamp held the sample at a fixed position (Fig. 1a). Its resistance was measured at > 1000 MΩ (overloaded) initially, using an isolation meter. Then electrical measurements were taken with a VersaSTAT potentiometer (VersaSTAT 4, Princeton Applied Research) under water evaporation. The specific surface area was measured using the Brunauer–Emmett–Teller (BET) method (Quantachrome, Autosorb-iQ). The alumina was characterized with an inductively coupled plasma optical-emission spectrometer (ICP-OES) (720-ES, Agilent) for Si, Al, Fe, Mg, Ca, Na, with an inferred absorption method after fusion (LECO TC-436Ar) under inert gas for O. The thermal conductometric method (LECO TC-436Ar) was used after fusion under inert gas for N and the infrared absorption method (LECO CS-844) was used after combustion for C. Fourier transform infrared (FTIR) spectra of alumina were recorded with a Nicolet iS50R (Thermo Scientific). Sixty-four scans were collected for each measurement in the spectral range of 400–4000 cm^−1^ with a resolution of 4 cm^−1^.

The 3.0 × 3.0 cm^2^ size alumina sample having 0.3 cm thickness was used (Fig. 1b). The measured specific surface area of the alumina via the BET was 1.5 cm^2^ mg^−1^. Inductively coupled plasma optical emission spectroscopy (ICP-OES) used to determine how much mass % of certain elements are present in alumina sample. The alumina was characterized by an ICP-OES (720-ES, Agilent) for Si, Al, Fe, Mg, Ca, Na and by an Inferred absorption method after fusion (equipment LECO TC-436Ar) under inert gas for O, Thermal conductometric method (equipment LECO TC-436Ar) after fusion under inert gas for N and Infrared absorption method (equipment LECO CS-844) after combustion for C. The chemical composition of alumina was confirmed as 47.3% Al, plus 1.44% Si, 0.03% Fe, 0.40% Mg, 0.02% Ca, 0.04% Na, 45% O, < 1% N and 0.056% C. Figure 1c shows the XRD pattern of alumina where characteristics peaks are attributed to the PDF card 10-0173 of α-alumina. Figure 1d shows the SEM image of alumina and Fig. 1e is the magnified view of Fig. 1d. The SEM image reveals that the alumina is composed of high-density, interconnected micropores and the 50–200 µm pore diameter is optimal for infiltrating water. The SEM/EDX was also used to investigate the composition of alumina (Figure S1a). The EDX spectrum (Figure S1b), indicates peaks of Al and O and Figure S1c-d present elemental mapping of Al and O.

FTIR spectra of alumina (Figure S2) show a dip with wavenumbers 3310 attributed to the –OH stretching mode and a dip at 1737 cm^−1^ that can be attributed to the bending mode^48^. The dip at 1074 cm^−1^ corresponds to Al–O–H and bands at 628, and 482 cm^−1^ are attributed to Al–O bonds^49^. Wettability and impregnation of water into the porous alumina were measured with a contact angle meter (DM700) and demonstrated (Figure S3) with a 1-µL water droplet placed on the alumina surface. Captured dynamic images of the droplet on the alumina illustrate a super hydrophilic surface. The inherently hydrophilic surface of alumina facilitates rapid flow of water through its pores, which greatly increases electricity generation driven by transpiration.

The alumina sample was connected with two electrodes and placed in a beaker to measure the potential drop across the sample. The alumina sample was inserted vertically into a beaker filled with deionized (DI) water covering one half of the alumina (1.5 cm), and leaving the upper half exposed to the atmosphere. One electrode placed at the end of the sample was immersed in water. The height to which water infiltrated was significantly higher than the water level in the beaker, due to the capillary action of the micropores. Mimicking transpiration in plants, with evaporation of water at the alumina block surface, water in the container had to be quickly replenished, infiltrating into the alumina above the water level.

## Results and discussion

Although porous alumina is an insulating material, it can be converted into a surface conductor when in contact with water and yields a substantial amount of electrical power associated with surface evaporation. The DI water used here has an electrical conductivity of 0.055 µS/cm, and the measured conductivity of completely wet alumina due to its water-dielectric charge interface was 1286.10 µS/cm (measured by zeta potential), orders of magnitude higher than that of pristine dry alumina (0.0001 µS/cm).

**Figure 2:**
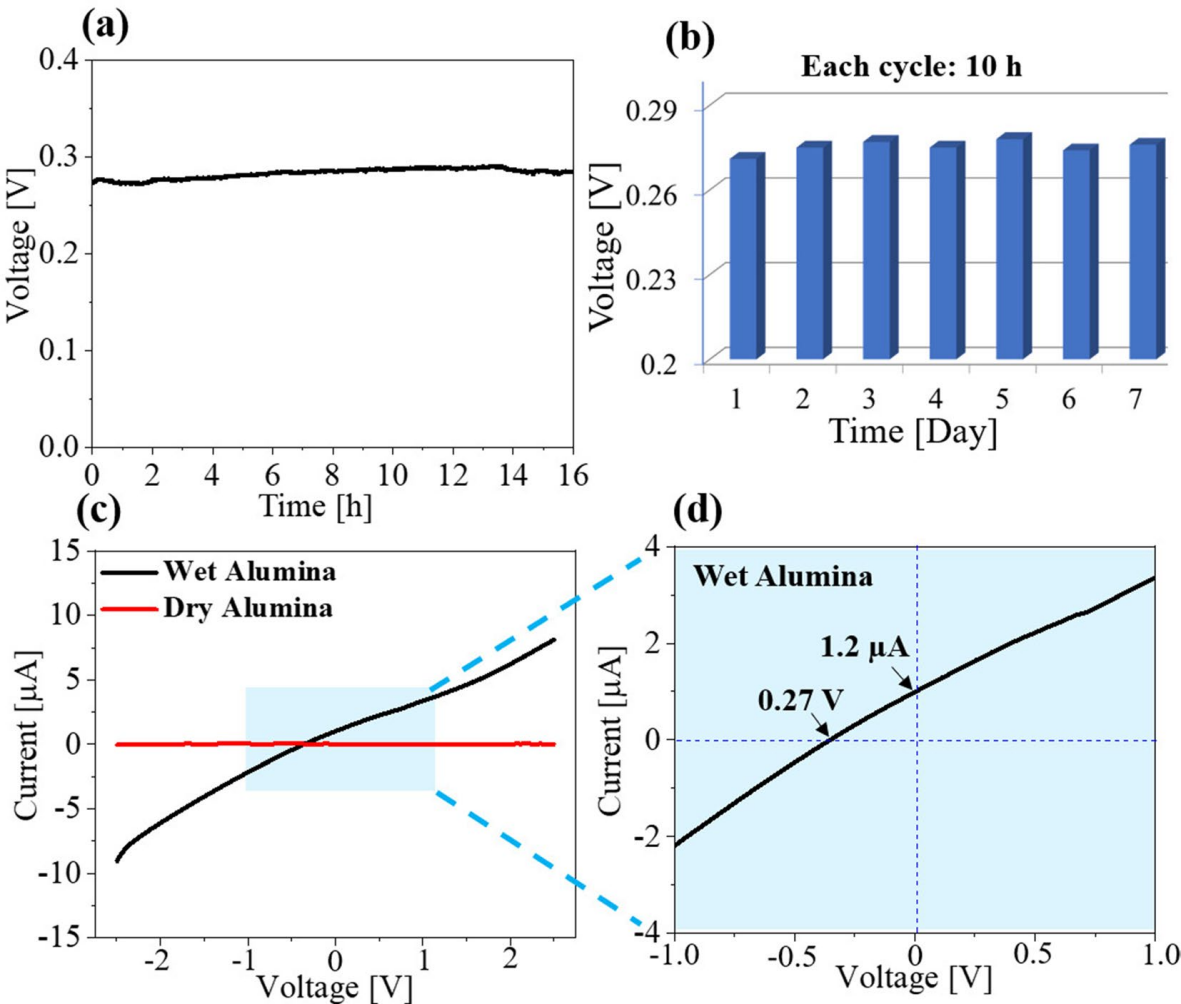
(**a**) Voltage generated by alumina with respect to time when the alumina block was immersed 1.5 cm in water. (**b**) Voltage generated by alumina continuously for 7 days, in which each cycle represents 10 h of measurements per day. (**c**) An I-V curve with the dry sample without water and wet sample half-immersed in water. (**d**) Magnified I-V curve near the origin shown in (**c**).

When the alumina block is partly soaked in water, voltage generation as high as 0.27 V was observed with high stability. The measured I-V curve exhibits a short circuit current of nearly 1.2 µA with an open-circuit voltage of 0.5 V. The alumina device shows good long-term stability (Fig. 2a) and required about an hour before achieving a stable voltage (Figure S4). Evaporation-induced voltage of the alumina device achieved a stable output of nearly 0.27 V after about 1 h under ambient conditions. To further check the long-term stability of alumina, the measurement was continued for 7 days, showing the stability of generated voltage (Fig. 2b). The I-V curves indicate that water-immersed alumina is conductive (Fig. 2c). A completely dry sample before addition of water shows 0 µA (Fig. 2c), as the resistivity is ~ 10^10^ Ω cm. After immersion in water, the IV curve exhibits a slope with an offset indicating the power-generating nature of the water/alumina system (Figs. 2c, d). Current versus time data are presented in Figure S5.

Maximum instantaneous output power density can be calculated^28^ as P_max_ = J_max_ × V_max_, where J_max_ (1.2 µA) and V_max_ (0.27 V) are the maximum short-circuit current density and open-circuit voltage, respectively. The maximum instantaneous output power density was calculated as 0.324 ± 0.02 µW. The observed phenomena resemble the behaviour of so-called streaming potential observed with candle black soot and carbon nanotubes in contact with water. However, in those cases, voltage values are an order of magnitude less than the current results and they are mechanically very fragile, because the material adheres only weakly to the substrate. In contrast, the present system is a rigid solid medium, and yields far higher voltage than reported carbon-based materials. This is rather surprising, as the original material is an electrical insulator when dry, yet it yields substantial voltage when in contact with water. As a control experiment, we prepared “*carbon-coated*” alumina using candle black soot (Figure S6) and other types of devices (Figure S7) and compared them with our pristine uncoated alumina. The result confirms that voltage generation is reduced significantly if the alumina surface is loaded with carbon, and other types of devices show far lower voltages (Note S1 and Note S2).

**Figure 3:**
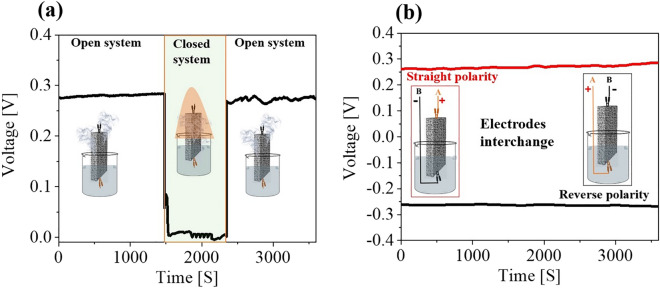
(**a**) Voltage signal in open and closed system with schematic representations. (**b**) Effect of electrodes interchange on induced voltage with sample setup schematics.

When the alumina was sealed within the beaker using a plastic wrapper (Figure S8), the induced voltage dropped, finally approaching to zero (Fig. 3a). When the beaker was sealed to create a closed system, water vapor content in the air above the water surface became remarkably high and evaporation eventually stopped. Therefore, it is apparent that electricity generation ceased when the saturation deficit reached zero. Moreover, evaporation-induced voltage of the alumina could be inverted and maintained at the same height, by flipping the two electrodes (Fig. 3b). When the device was inverted and the other end was immersed in water without changing the electrical connections, the voltage reversed its sign, but reached the same amplitude. These results indicate that water evaporation is the driving force for the electricity generation, and that the direction of the voltage is correlated with the direction of water flow driven by evaporation.

**Figure 4:**
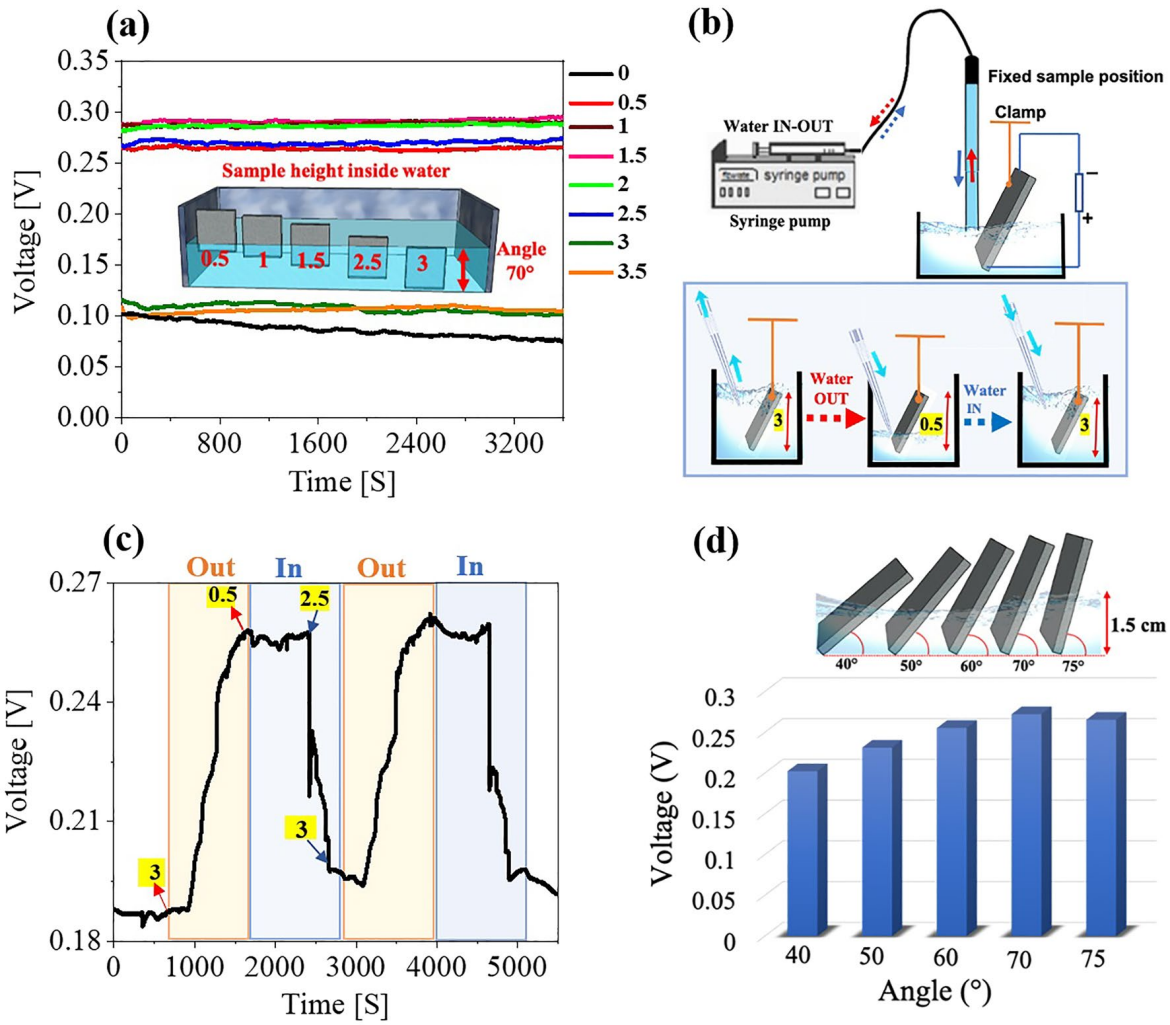
Voltage generation performance of alumina with (**a**) different sample heights inserted into the water, where the key shows different degrees (cm) of sample immersion in water and the inset schematic presents the amount of sample immersion in water at 70°. (**b**) Schematic representation of sample height (cm) in the water. (**c**) Dependence of the induced voltage difference on water transpiration speed. (**d**) Different sample placement angle at 1.5 cm sample height in water.

Further insights into voltage generation can be obtained from additional experiments using DI water with the alumina sample immersed to different extents in water. Maximum voltage was observed when the alumina was 1, 1.5 or 2 cm inserted into water (Fig. 4a). Generated voltage was reduced after inserting the sample 0.5 or 2.5 cm into the water, where either electrode was at the water’s surface. The voltage diminished further when the sample was completely in or out of the water. Placing the air–water interface in the middle of the porous alumina sample was essential for voltage generation, which effectively promoted evapotranspiration through the porous media.

After that, the behaviour of the induced voltage was examined with water ejection and injection with a speed of 100 µL/S and a 70° placement angle. A syringe pump was used (Fig. 4b). The beaker was initially filled to 3 cm sample height, after few minutes when the water was gradually ejected from the beaker until 0.5 cm sample height, voltage quickly increased and became maximal (Fig. 4b, c). Afterward, water was re-filled, nearly reaching the 3-cm sample height (yet partly exposed to the air) and voltage decreased to 0.18 V from 0.26 V. This behaviour was highly reproducible. When the voltage difference reached its maximum and remained stable, an equilibrium state was attained for flowing water molecules inside the alumina, i.e., when some water molecules outflow at one end the same number of water molecules entered the alumina from the other end. However, when the water was re-filled beyond 2.5 cm on the sample and immersed nearly completely, the equilibrium was disturbed due to a quick reduction of the air-alumina interface; therefore, the voltage dropped very quickly. Similar voltage behaviour was also observed for streaming potential in carbon nanotubes^23^.

Next, streaming voltage performance was examined with faster water supply and removal rates (250 and 500 µL/S) from the beaker (Figure S9a-b). From this result we can see that the voltage generation is also affected by the dynamic movement of the water level in the beaker. Then, the influence of sample placement angle on voltage generation was also investigated, keeping the sample height at 1.5 cm in water (Fig. 4d). Maximum voltage was observed at a sample angle of 70°.

**Figure 5:**
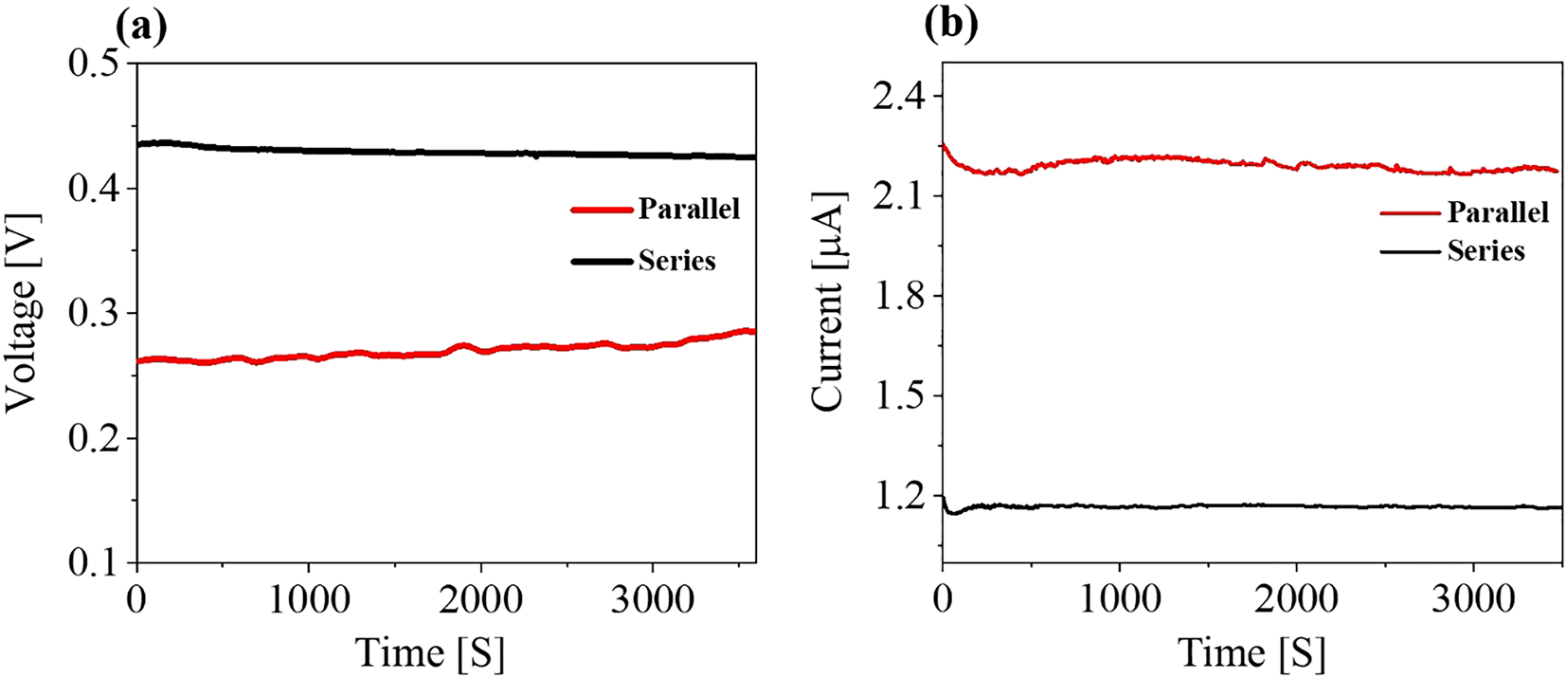
(**a**) Voltage and (**b**) Current generated by two alumina samples in series and parallel connections.

It should be noted that output performance of the alumina device can be easily scaled up with simple series or parallel connections. Two alumina samples were connected in series and in parallel (Fig. 5a, b). When samples were connected in parallel, the voltage was the same as that of a single sample (Fig. 2a); however, the current nearly doubled during current measurements. When two alumina samples were connected in series and parallel, the corresponding open circuit voltage and short circuit values accorded with series and parallel circuit law (Fig. 5b). Therefore, the alumina device can be boosted to any value by connecting device units in series and in parallel when more electricity is needed and can be used as a stable power source in electronic devices. One the other hand, by changing the alumina size we did not observed any significant difference in the induced voltage (Note S3; Figure S10).

**Figure 6:**
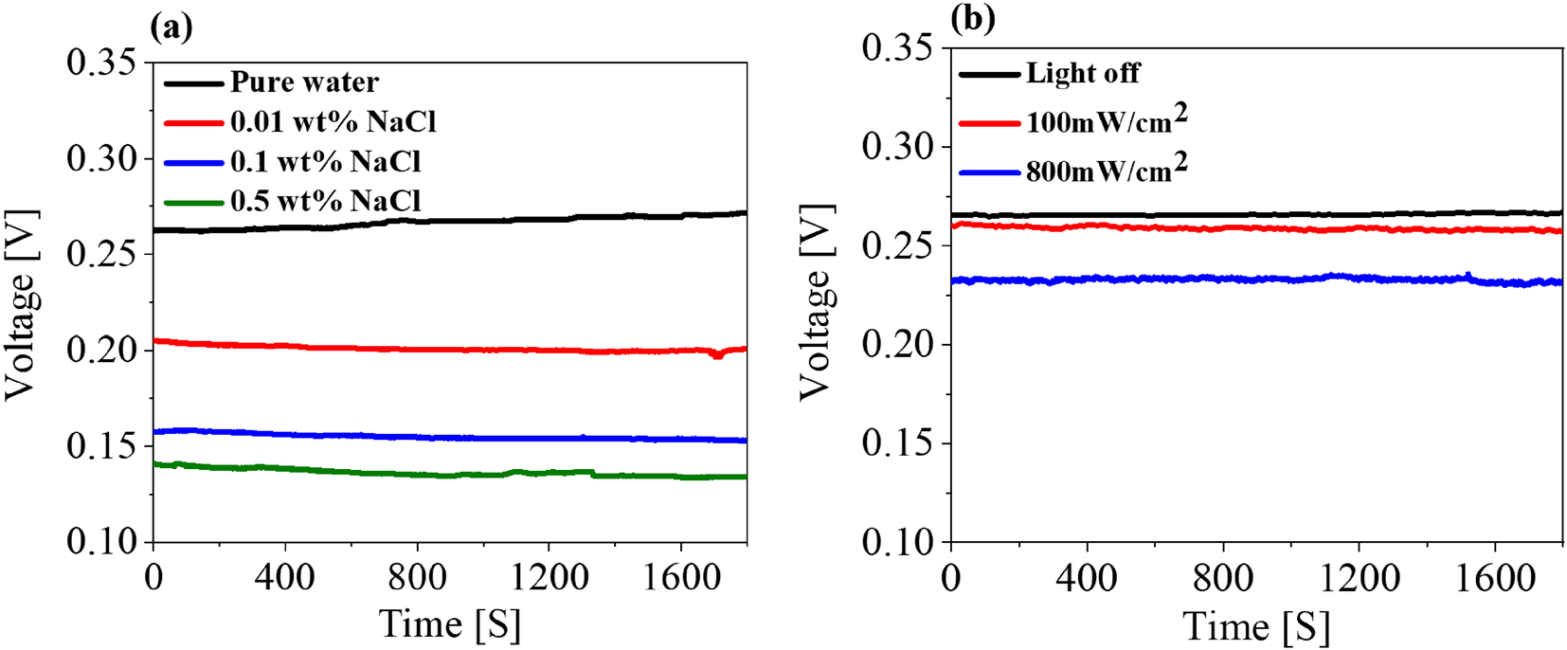
Voltage generation in relation to (**a**) NaCl concentration and (**b**) simulated sunlight intensity.

Dependence of the generated voltage on the ionic concentration was also studied to examine whether intrinsic properties of the liquid affect the performance of the water/ alumina interface. Three concentrations of NaCl, 0.01, 0.1 and 0.5 weight percentage (wt.%) were compared with DI water. All salt solutions produced much lower voltages than DI water (Fig. 6a). The Debye length, which is the distance for significant charge separation to occur, is inversely proportional to the square root of the ionic concentration^50, 51^. Above the critical molar concentration, an increase in the number of anions screens the polarization at the alumina/water interface, possibly redistributing the surface charge accumulated at the alumina/water interface. Therefore, the power generation capacity of the alumina device in the above three NaCl solutions changes significantly with salt concentration.

Next, the effect of illumination on voltage generation was investigated by illuminating the alumina block with high-intensity (800 mW/cm^2^) and standard (100 mW/cm^2^) simulated sunlight using a solar simulator. The voltage decreased as solar simulator irradiance increased (Fig. 6b). During illumination, alumina absorbed light and due to photothermal heating, the wet alumina surface dried, partly suppressing the amount of water moving upward. Initial 1-h values of voltage generation plotted against time for the alumina sample of NaCl concentration and light intensity as shown in Figure S11.

**Figure 7:**
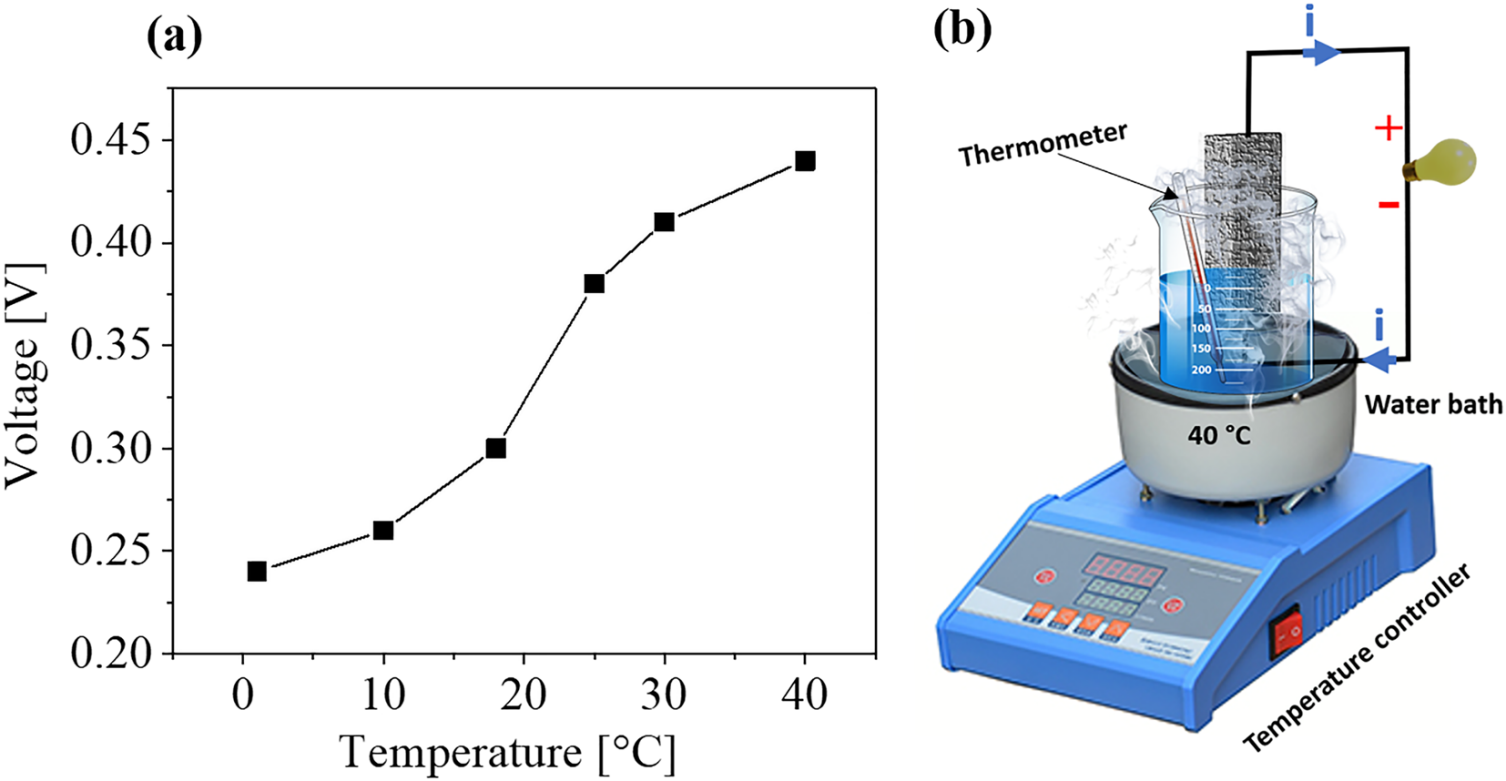
(**a**) Evaporation-induced voltage plotted as a function of temperature; (**b**) Schematic representation of water transpiration through alumina.

Since wetting of the alumina surface and water evaporation are essential to this power generation process, we further examined the effect of temperature. Figure 7 shows the voltage signals as the water temperature was varied from 1 to 40 °C in a temperature-controlled water bath. A larger electric signal was detected at higher temperatures (Figs. 7 and S12). Since vaporization of water is enhanced at higher temperatures, water flow at the alumina interface is increased, enhancing power generation. These results suggest the utility of this method to produce electricity in warmer environments. Such alumina-based, evaporation-induced electrical generation enables a new means of converting waste heat into electricity. Since alumina is a rather stable and widely used industrial ceramic, this evaporation-induced energy harvesting can open a new field in ceramic research, utilizing porous materials for small-scale energy harvesting devices for circumglobal applications.

**Figure 8:**
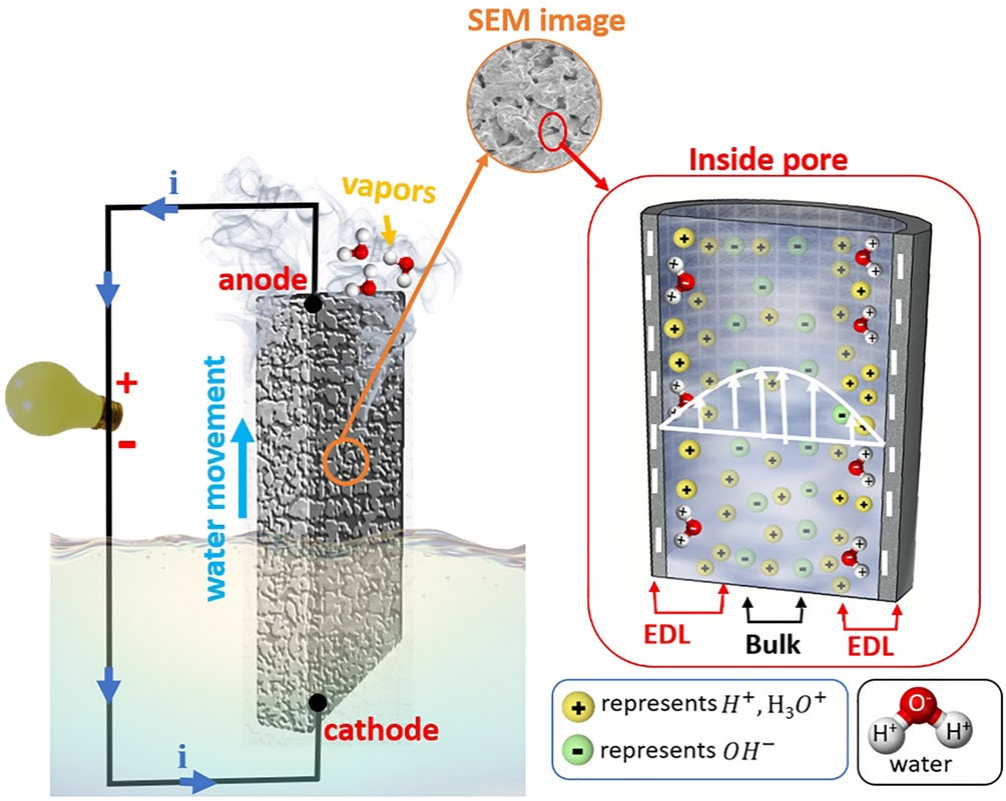
Schematic diagram of evaporative power generation.

We further discuss the possible mechanism of evaporation-induced power generation at the alumina surface. First, the zeta potential of the alumina was measured at − 98.19 mV (Figure S13). The zeta potential gives an indication of the negative charge present on the alumina surface^52^ and this negative charge may be associated with surface –OH groups on the alumina surface, consistent with our FTIR measurements (Figure S2)^49^. These –OH functional groups exhibit a high negative zeta potential on the surface and make the interior of the porous alumina super hydrophilic. Water molecules are attracted to the hydroxylated alumina surface, wetting it and then moving upward through the porous channels via capillary action, until they eventually evaporate (Fig. 8). Protons (H^+^), or hydronium ions (H_3_O^+^) accumulate along the alumina surface and form a polarized surface layer known as the electric double layer (EDL), attracted by the negative surface charge. The thickness of the EDL is relatively large (several hundreds of nanometers) due to the large Debye screening length of pure water. In this EDL region, a substantial numbers of protons are attracted to the water alumina interface. Then a deficiency of protons in the channel centers needs to be compensated by additional water self-ionization, which subsequently boosts the conductivity of the entire system^53, 54^. These ions in the pore-confined water freely flow upward along the channel due to transpiration. Contrarily, protons trapped directly on the surface or captured in the interfacial water network adsorbed on the surface can hop or move moderately via a Grotthuss mechanism^55, 56^. Accordingly, these ions migrate uphill together with water under a capillary force as water evaporates from the alumina; thus, the above-described interfacial charges (positive and negative) are also dragged simultaneously in the same direction. Since their distribution near the surface region differs, positive net current is generated due to the possibility of different diffusion speeds for two the components. Then the two electrodes attached to the upper and lower sides can monitor the spontaneous voltage generation across the entire alumina sample. Together with this, flow charge dynamics in transpiring water, other possible causes, such as the chemical potential difference of the top and the bottom electrodes may arise from the difference in their environments (air versus water). Since the alumina/electrode system is symmetric in its geometry, voltage generation across this symmetric device must either originate from the uniaxial movement of water or the amount of oxygen in contact to the two electrodes. This can contribute to reduction of the stainless clamp, which seems to be a minor contribution, since the change in pH value of water was minute (~ 0.8) even after one week of continuous measurement.

## Conclusion

We have demonstrated an extremely simple method of electrical generation using microporous ceramic blocks partly soaked in water. The evaporation-driven movement of water through micropores of microporous alumina generates electricity, due to dragging of carriers at the water-alumina interface. The microporous alumina is highly hydrophilic and contains numerous pores with a large specific surface area that contributes to rapid water flow and formation of near-surface carriers. Evaporation-driven water flow within porous alumina membranes can generate stable voltage up to 0.27 V under ambient conditions. Our results show that the amount of power generated can be increased by elevating the water temperature. The current results inspire us to apply inexpensive alumina to produce electricity in warm, dry environments. The significance of this work is that porous alumina not only offers an inexpensive and efficient small-scale power generator, but also opens a new way to harvest energy by water transpiration, which should be feasible any place at any time on earth as long as there is water in liquid form.

## Supplementary Information

Supplementary Information.
