# Supplementary material for: Hydropower generation by transpiration from microporous alumina
Source: Sci Rep. 2021 May 26;11:10954. doi: 10.1038/s41598-021-90374-5 (PMC8155211; doi:10.1038/s41598-021-90374-5)
Supplement: Supplementary file 1 — Supplementary Information. [file 41598_2021_90374_MOESM1_ESM.docx]

**Supporting Information**

**Hydropower Generation by Transpiration from Microporous Alumina**

Manpreet Kaur^a^, Satoshi Ishii^a^, Ryusuke Nozaki^b^, and Tadaaki Nagao^a,b^

^a^International Center for Materials Nanoarchitectonics (WPI- MANA), National Institute for Materials Science (NIMS), Tsukuba, Ibaraki, 305-0044, Japan

^b^Department of Condensed Matter Physics, Hokkaido University, Sapporo, Hokkaido, 060-0810, Japan

**Table of contents: (Total pages. 10; 13 figures)**

1. SEM/EDX characterization of alumina showing (a) bright-field image, (b) EDX spectrum, (c) and (d) elemental mappings of Al and O. Each scale bar represents 100 µm.

2. (a) FTIR spectrum of alumina. (b) and (c) show magnified views of (a).

3. Time sequence photos when 1 μL of water was dropped on the alumina sample. The photo shows that alumina is hydrophilic.

4. Initial 1-hour values of voltage generation plotted against time for the alumina sample.

5. Time dependent current of the alumina sample.

6. (a) Comparison of voltage generation performance of alumina and carbon coated alumina (b) SEM image of carbon coated alumina (inset. digital image).

7. Comparison of different kinds of black and white microporous alumina and their induced voltages. Scale bars in SEM images represent 100 µm.

8. Photographs of alumina in (a) open and (b) closed beakers containing water.

9. Streaming voltage performance variation (a) with 250 and (b) 500 µL/S quantity of water ejected and injected into the beaker.

10. (a) Dependence of the induced voltage difference on the sample size and illumination. (b) Photograph of samples.

11. Time dependence of the generated voltage with (a) different NaCl concentration and (b) artificial sunlight intensity. After about 1-hour of electricity generation, voltage stabilizes, regardless of NaCl concentration and light intensity.

12. Induced voltage as a function of temperature.

13. Zeta potential of the alumina.


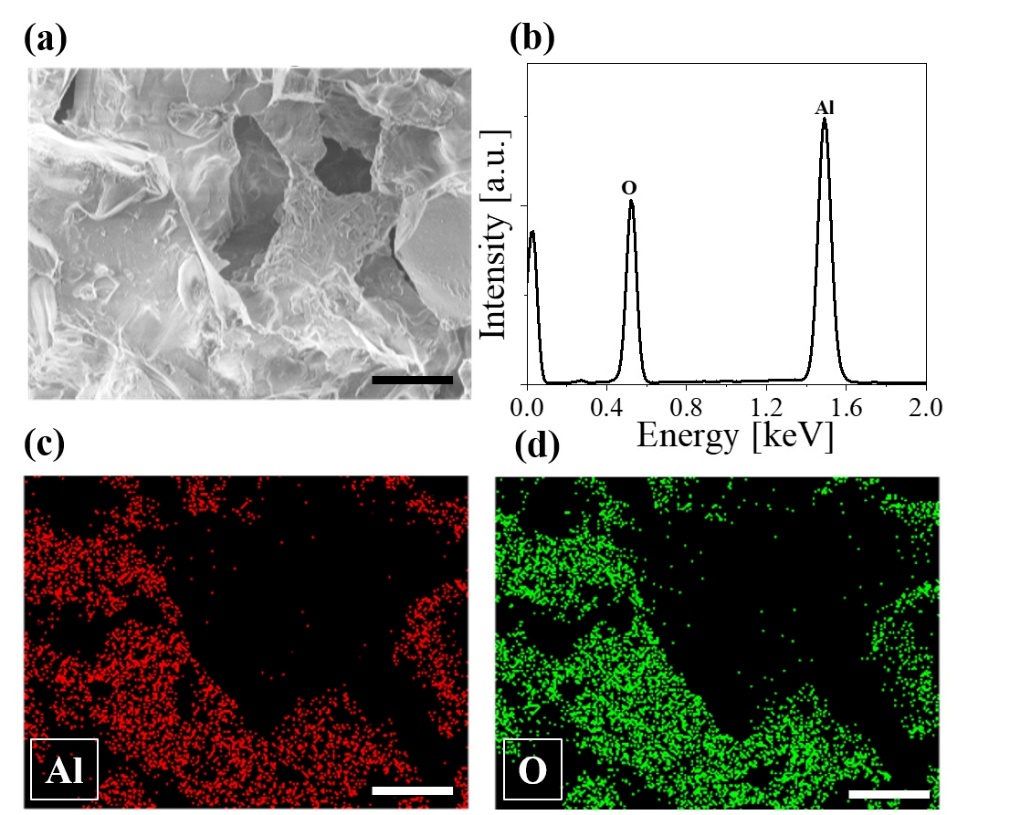


**Figure S1.** SEM/EDX characterization of alumina showing (a) a bright-field image, (b) an EDX spectrum, (c) and (d) elemental mappings of Al and O. Each scale bar represents 100 µm.


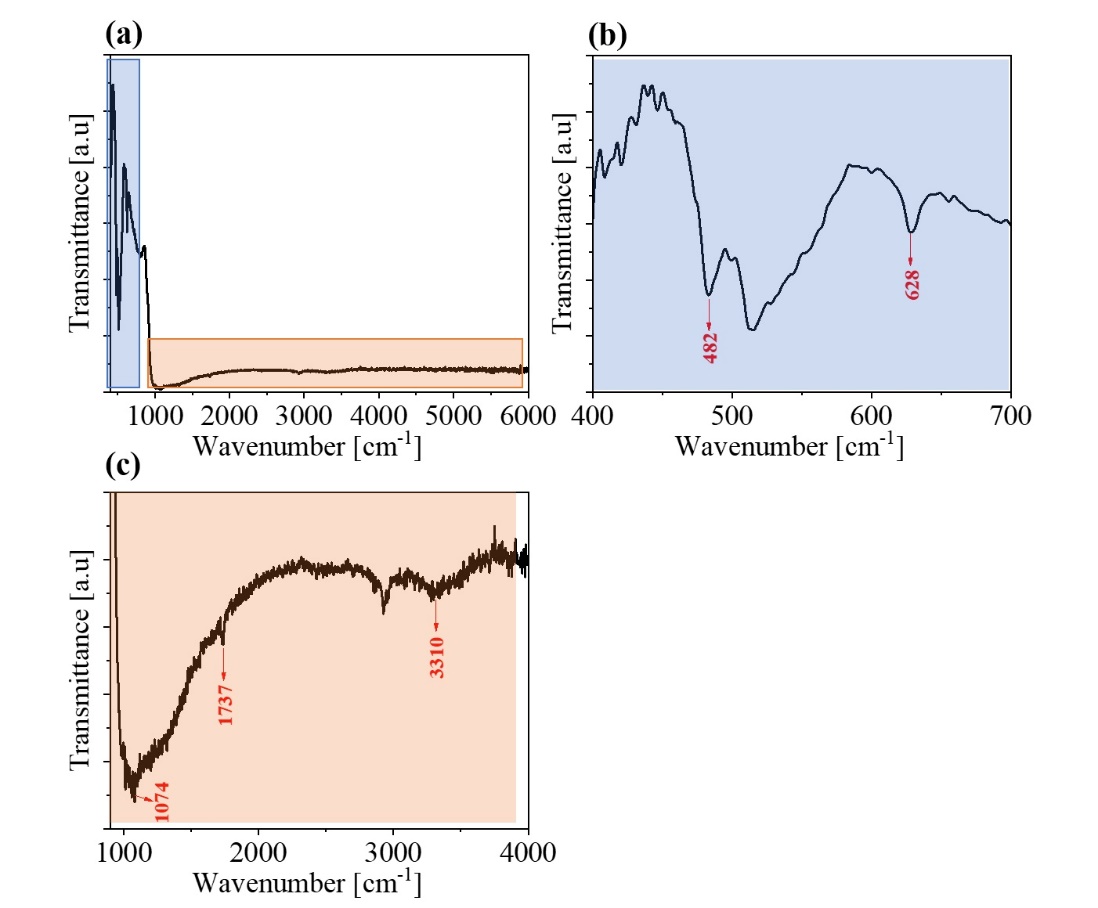


**Figure S2.** (a) FTIR spectrum of alumina. (b) and (c) show magnified views of (a).


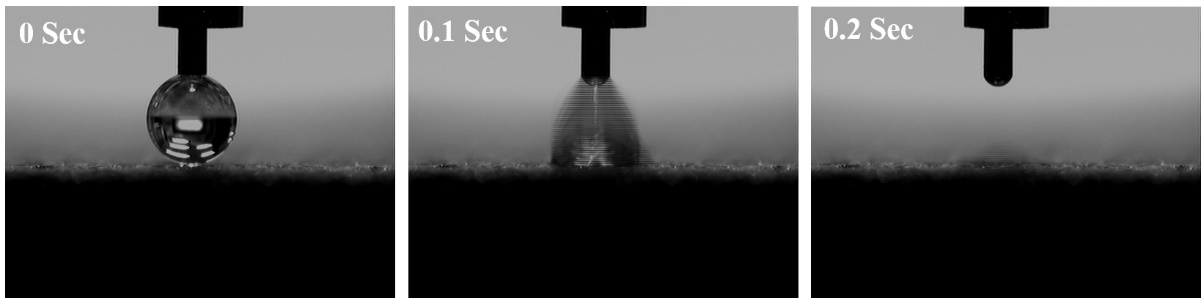


**Figure S3.** Time sequence photos when 1 μL of water was dropped on the alumina sample, showing that alumina is hydrophilic.


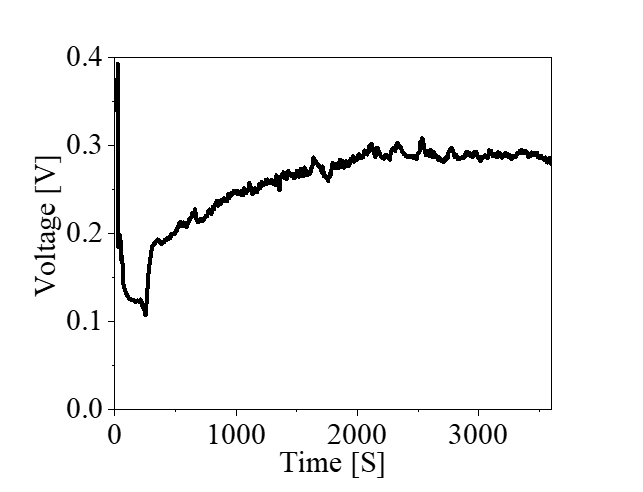


**Figure S4.** Initial 1-hour values of voltage generation plotted against time for the alumina sample.


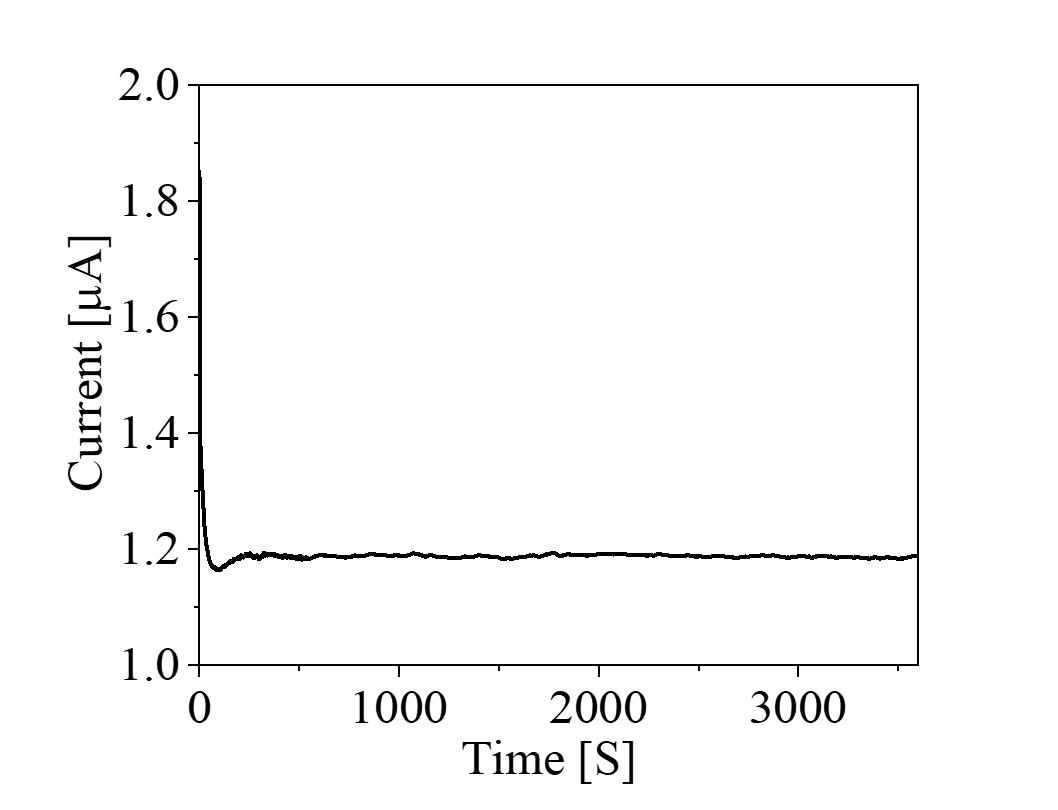


**Figure S5.** Time-dependent current of the alumina sample.

**Note S1.** As a control experiment on the conductive system, the output voltage for *“carbon-coated”* alumina was compared with uncoated alumina (Figure S6a). A significant decrease in voltage output was observed with carbon coating, which can be attributed to short circuiting through the carbon layer with lower resistance. In fact, the overall morphology of carbon-coated alumina reveals that a large number of carbon particles are coated on the alumina and percolate at the alumina surface (Figure S6b). Accordingly, the system behaves like previously reported carbon-based systems. Here, electricity generation occurs predominantly at the alumina-liquid interface, while the carbon coating forms a current path with lower resistance, which forcibly reduces the output voltage. Hence, uncoated alumina is preferable for voltage generation in comparison with carbon surfaces.


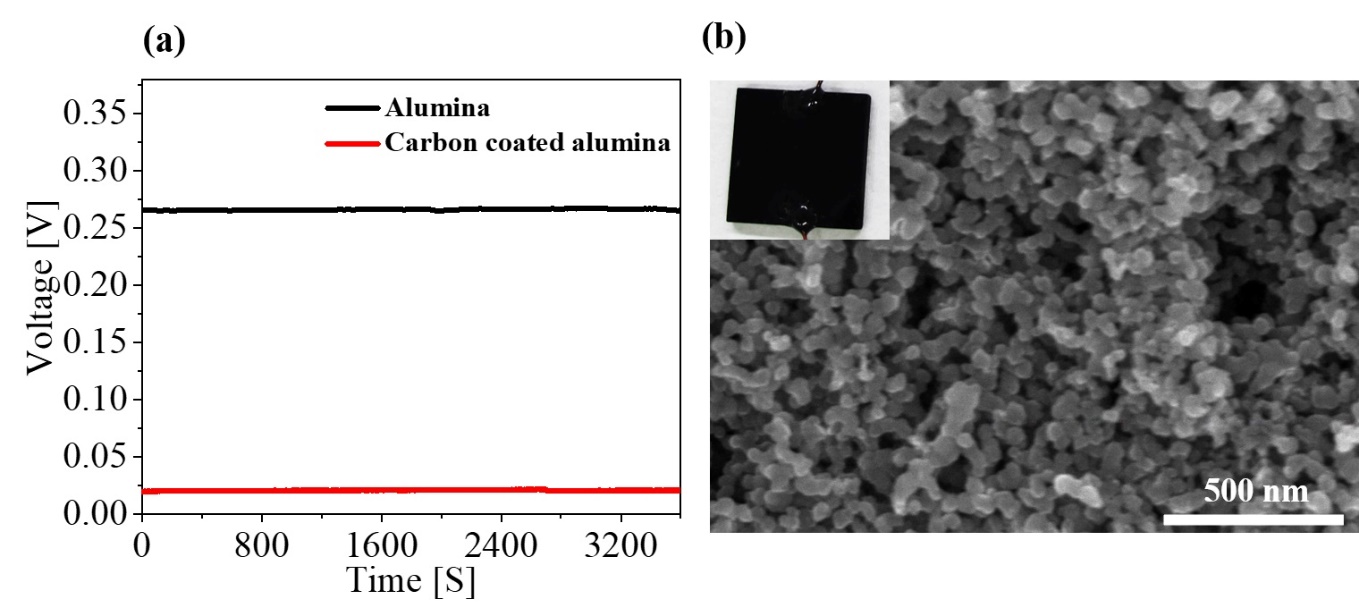


**Figure S6.** (a) Comparison of voltage generation performance of alumina and carbon coated alumina (b) SEM image of carbon coated alumina (inset. digital image).

**Note S2.** To confirm that this phenomenon also occurs with different types of alumina, we investigated induced voltage from another type of alumina product (a *white one* from the same company) in addition to the one shown in Figure 1 (*black*). The SEM, top view and side view optical images taken in the contact angle measurement system of other porous alumina are presented in Figure S7. Alumina samples (a) and (b) have higher induced voltages, due possibly to their larger pore diameters (50 ~ 200 µm) than samples (c) and (d), promoting faster water transpiration. Samples (c) and (d), which have smaller pores, infiltrate water very slowly, as its clear from their contact angle measurements; therefore, induced voltage generation performance is low. Also, samples (a) and (b) have shown durable performance, which is essential for practical applications. Therefore, we chose sample (a) for all measurements.

Thus, the alumina with higher porosity and larger contact angle enhances the water–material interaction and shows the larger voltage generation. The water evaporation induced by the hydrodynamic pressure gradient can transport more counterions between top and bottom electrodes in 50 ~ 200 µm alumina pores than in smaller pores, which establish a large electrical potential difference between two ends of the porous alumina medium. However, its limited output and characteristics can be improved by future studies on developing electric double layer model for alumina pores size effect on water–material interaction.

| **SEM images of alumina samples**  **(Scale bar represents 100 µm)** | **Top view images & side view image taken with a contact angle imaging system** | **Parameters** |
| --- | --- | --- |
| 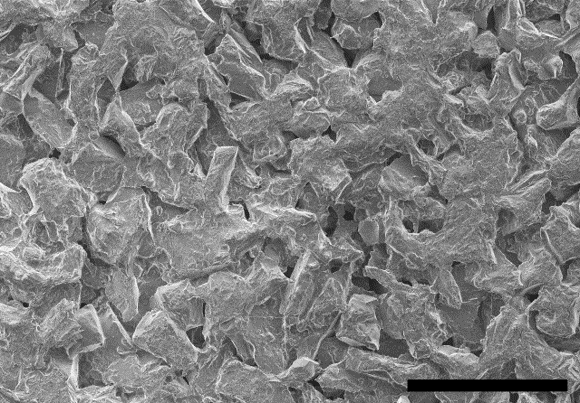(a) | 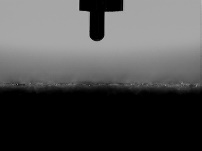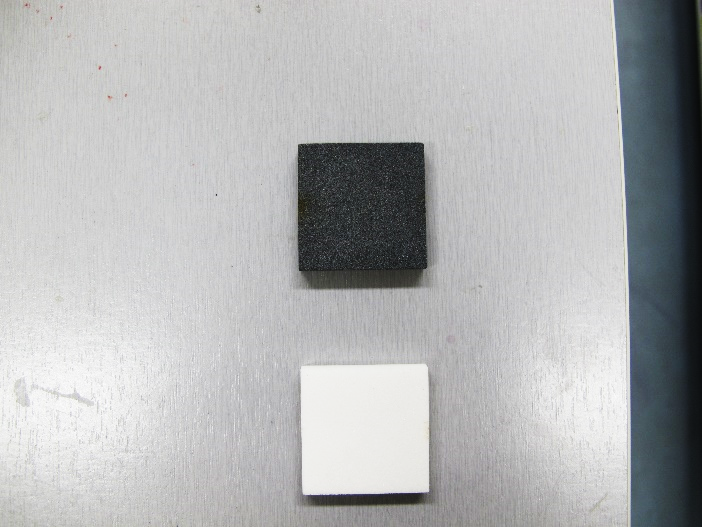 | Pore diameter: 50 ~ 200 µm  Induced Voltage: ~0.27V  Sample size: 3 × 3 cm^2^  Contact angle: 0°  Sample absorb water within a second.  (This sample was used for all measurements) |
| 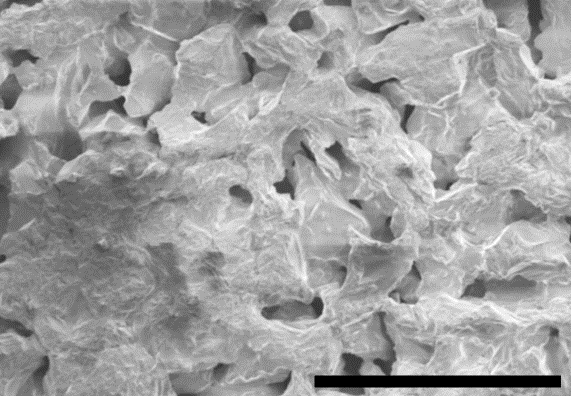(b) | 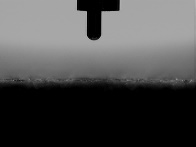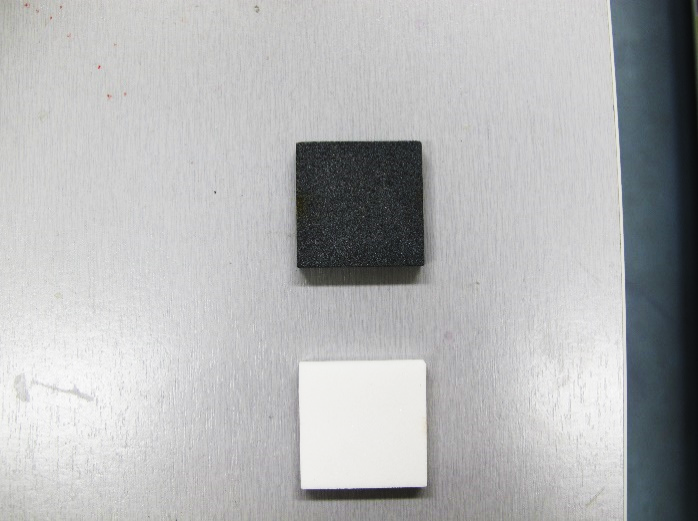 | Pore diameter: 50 ~ 100 µm  Induced Voltage: ~0.26V  Sample size: 3 × 3 cm^2^  Contact angle: 0°  Sample absorb water within a second. |
| 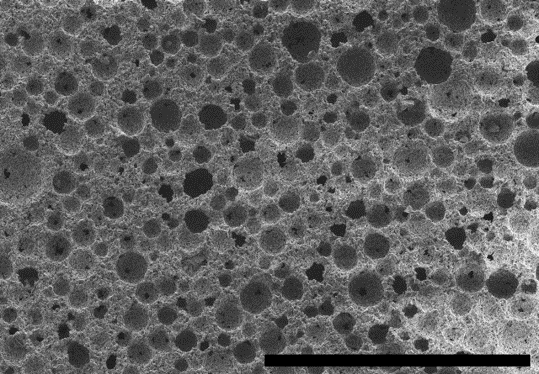(c) | 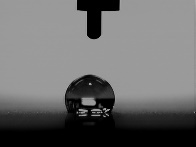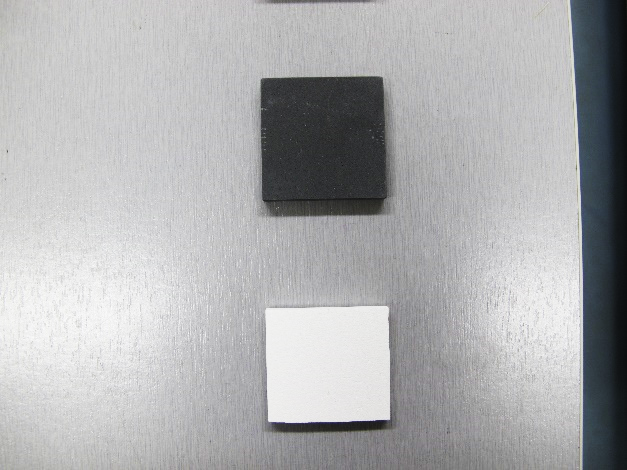 | Pore diameter: 40 ~ 50 µm  Induced Voltage: ~0.125V  Sample size: 3 × 3 cm^2^  Contact angle: 120°  Sample absorb water in 5 seconds. |
| 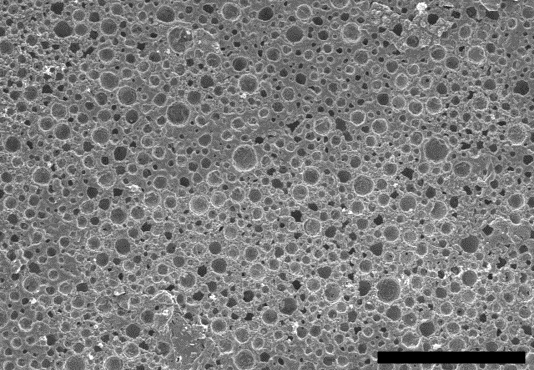(d) | 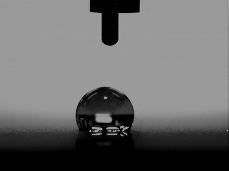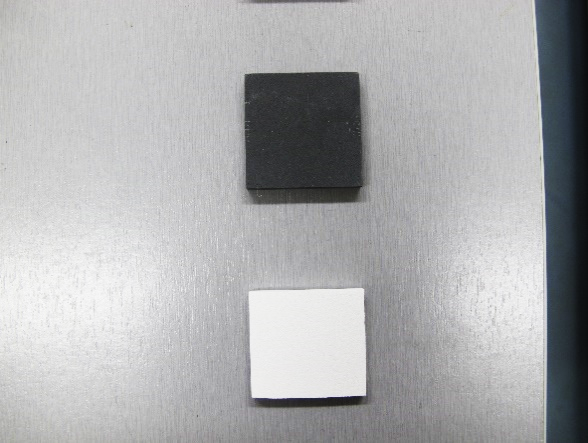 | Pore diameter: 40 ~ 50 µm  Induced Voltage: ~0.125V  Sample size: 3 × 3 cm^2^  Contact angle: 120°  Sample absorb water 5 seconds. |

**Figure S7**. Comparison of different kinds of microporous alumina and their induced voltages (a) black alumina, pore diameter: 50 ~ 200 µm (b) white alumina, pore diameter: 50 ~ 100 µm (c) black alumina, pore diameter: 40 ~ 50 µm (d) white alumina, pore diameter: 40 ~ 50 µm. Scale bars in SEM images represent 100 µm.

***
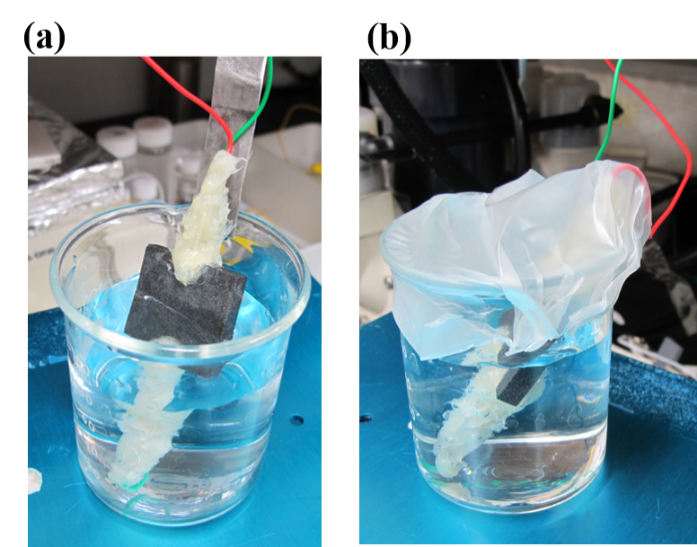
***

**Figure S8.** Photographs of alumina in (a) open and (b) closed beakers containing water.

**
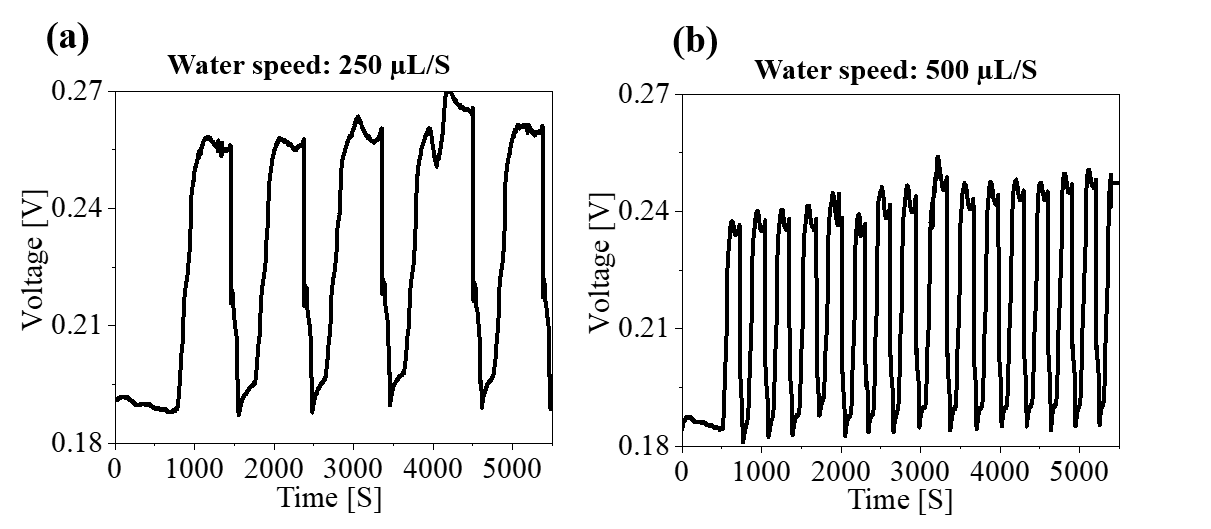
**

**Figure S9**. Streaming voltage performance variation (a) with 250 and (b) 500 µL/S of water ejected and injected into the beaker.

**Note S3.** Other factors that influence alumina power generation were tested by selecting different sample lengths. We employed 6×3 cm^2­^ and 9×3 cm^2^ samples and we measured their voltage difference by half-immersing them in the water. All samples generated voltages similar to the 3×3 cm^2^ sample, (Figure S10), when water infiltrated the alumina. We observed that changing the sample length did not enhance the alumina performance, seemingly because the amount of water reaching the top side of the alumina electrode is similar in all of the above cases, as can be judged by the wettability of the samples. To modify wetting/infiltration conditions of the samples, when 800 mW/cm^2^ solar light is applied near the top side electrode, it reduces the induced voltage. This is similar to the case of the 3×3 cm^2^ sample (Figure 6b). This phenomenon is associated with partial drying of the samples, which reduces the amount of water reaching the top.


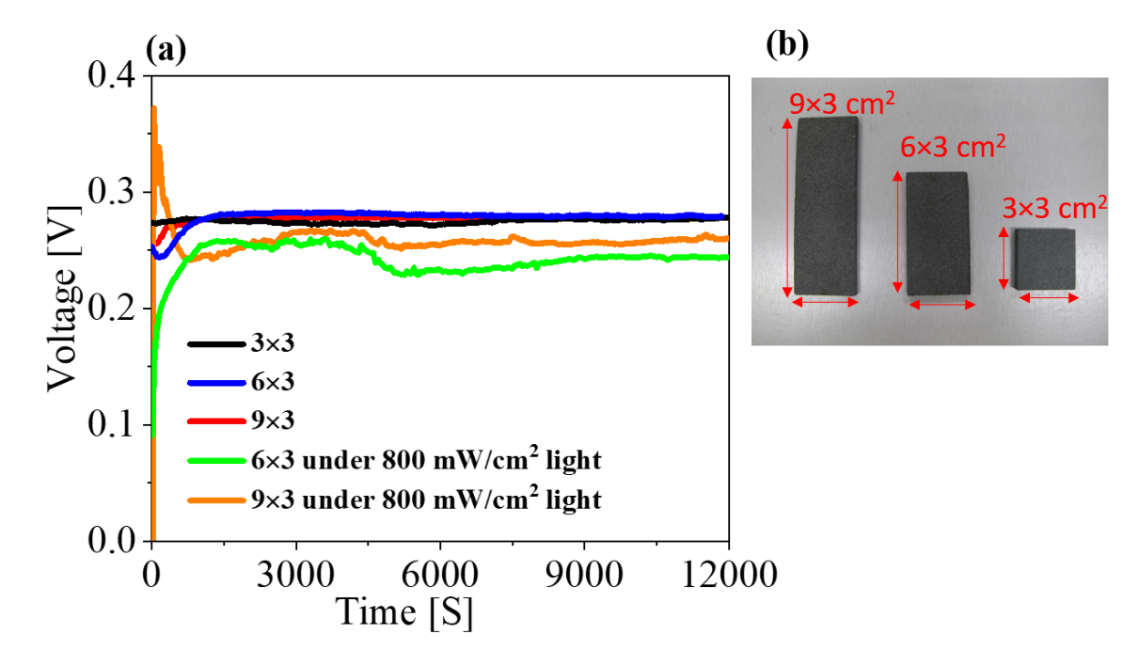


**Figure S10.** (a) Dependence of the induced voltage difference on the sample size and illumination. (b) Photograph of samples.

**
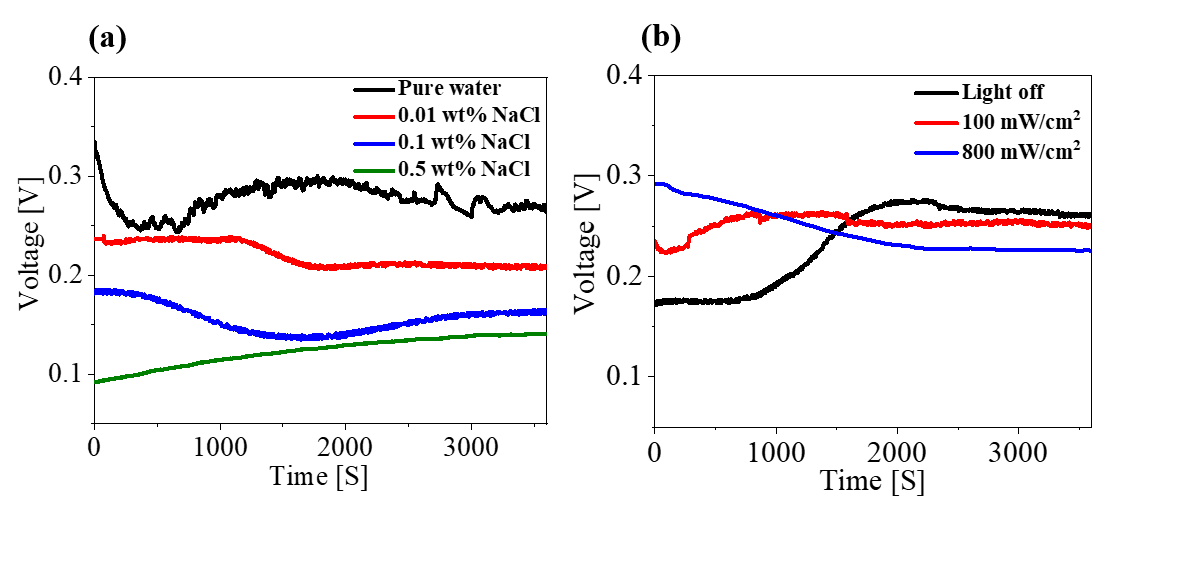
**

**Figure S11**. Time-dependence of the generated voltage with (a) different NaCl concentrations and (b) artificial sunlight intensity. After about 1 hour of electricity generation, voltage stabilizes, regardless of NaCl concentration and light intensity.

**
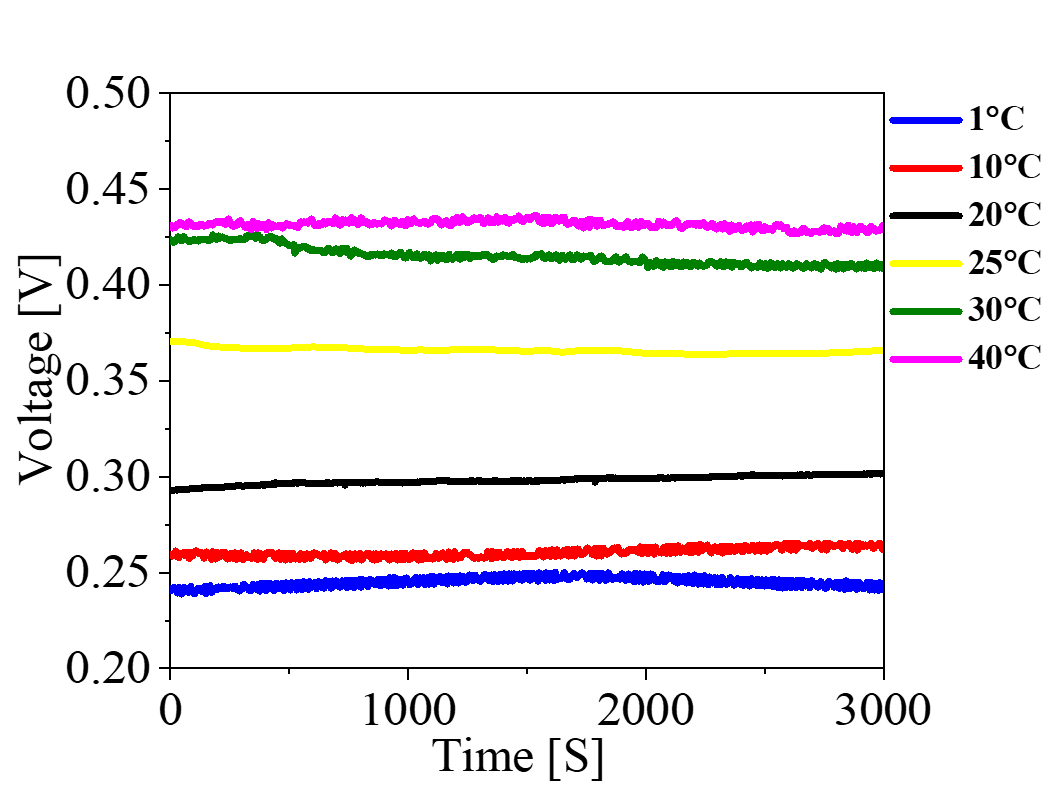
**

**Figure S12.** Induced voltage as a function of temperature.


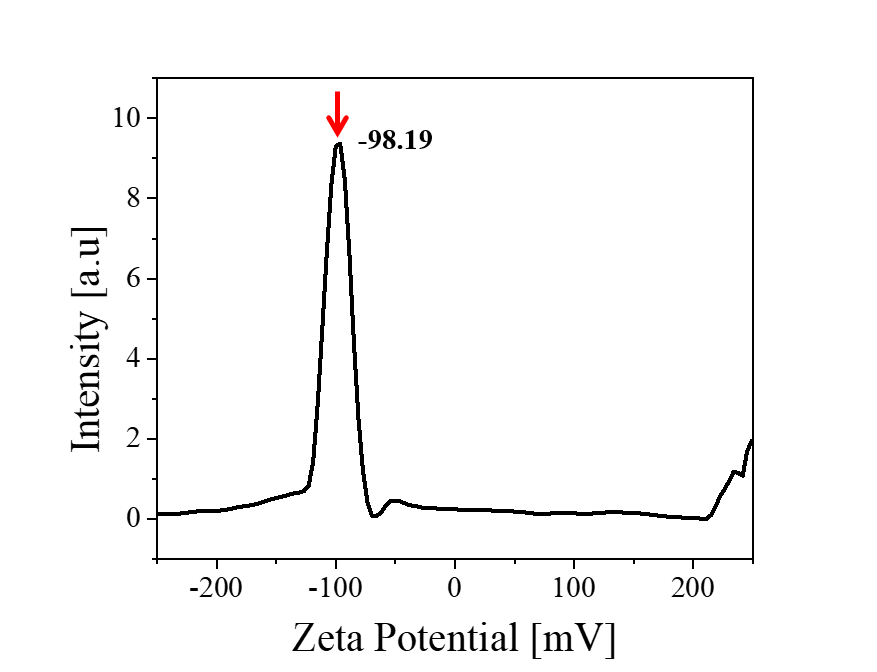


**Figure S13.** Zeta potential of the alumina.
